# Achieving Peptide Binding Specificity and Promiscuity by Loops: Case of the Forkhead-Associated Domain

**DOI:** 10.1371/journal.pone.0098291

**Published:** 2014-05-28

**Authors:** Yu-ming M. Huang, Chia-en A. Chang

**Affiliations:** Department of Chemistry, University of California Riverside, Riverside, California, United States of America; Russian Academy of Sciences, Institute for Biological Instrumentation, Russian Federation

## Abstract

The regulation of a series of cellular events requires specific protein–protein interactions, which are usually mediated by modular domains to precisely select a particular sequence from diverse partners. However, most signaling domains can bind to more than one peptide sequence. How do proteins create promiscuity from precision? Moreover, these complex interactions typically occur at the interface of a well-defined secondary structure, α helix and β sheet. However, the molecular recognition primarily controlled by loop architecture is not fully understood. To gain a deep understanding of binding selectivity and promiscuity by the conformation of loops, we chose the forkhead-associated (FHA) domain as our model system. The domain can bind to diverse peptides via various loops but only interact with sequences containing phosphothreonine (pThr). We applied molecular dynamics (MD) simulations for multiple free and bound FHA domains to study the changes in conformations and dynamics. Generally, FHA domains share a similar folding structure whereby the backbone holds the overall geometry and the variety of sidechain atoms of multiple loops creates a binding surface to target a specific partner. FHA domains determine the specificity of pThr by well-organized binding loops, which are rigid to define a phospho recognition site. The broad range of peptide recognition can be attributed to different arrangements of the loop interaction network. The moderate flexibility of the loop conformation can help access or exclude binding partners. Our work provides insights into molecular recognition in terms of binding specificity and promiscuity and helpful clues for further peptide design.

## Introduction

Signal transduction and DNA repair in the cellular communication pathway require specific molecular recognition to engage upstream and downstream regulation [1]–[5]. The binding mechanism is usually mediated by modular domains, which are precise and proficient in selecting particular motifs among a broad range of associates [6]–[9]. For example, one class of signaling domains, including Src homology 2 (SH2), breast cancer 1 (BRCA1) C-terminus (BRCT), forkhead-associated (FHA) and WW domain, can recognize phosphoproteins for functional specificity [10]–[14]. In contrast to the binding selectivity of phosphorylated sequences, signaling domains can interact with various partners with similar affinity, called binding promiscuity [15]–[19]. This observation raises an interesting question of how the signaling domains show both specificity and promiscuity during the recognition process. In this study, we chose the FHA domain as a model system to address this question. Although the FHA domain is an absolute phosphothreonine (pThr) binding module, it can efficiently bind to diverse peptide sequences [20]–[23].

**Figure 1:**
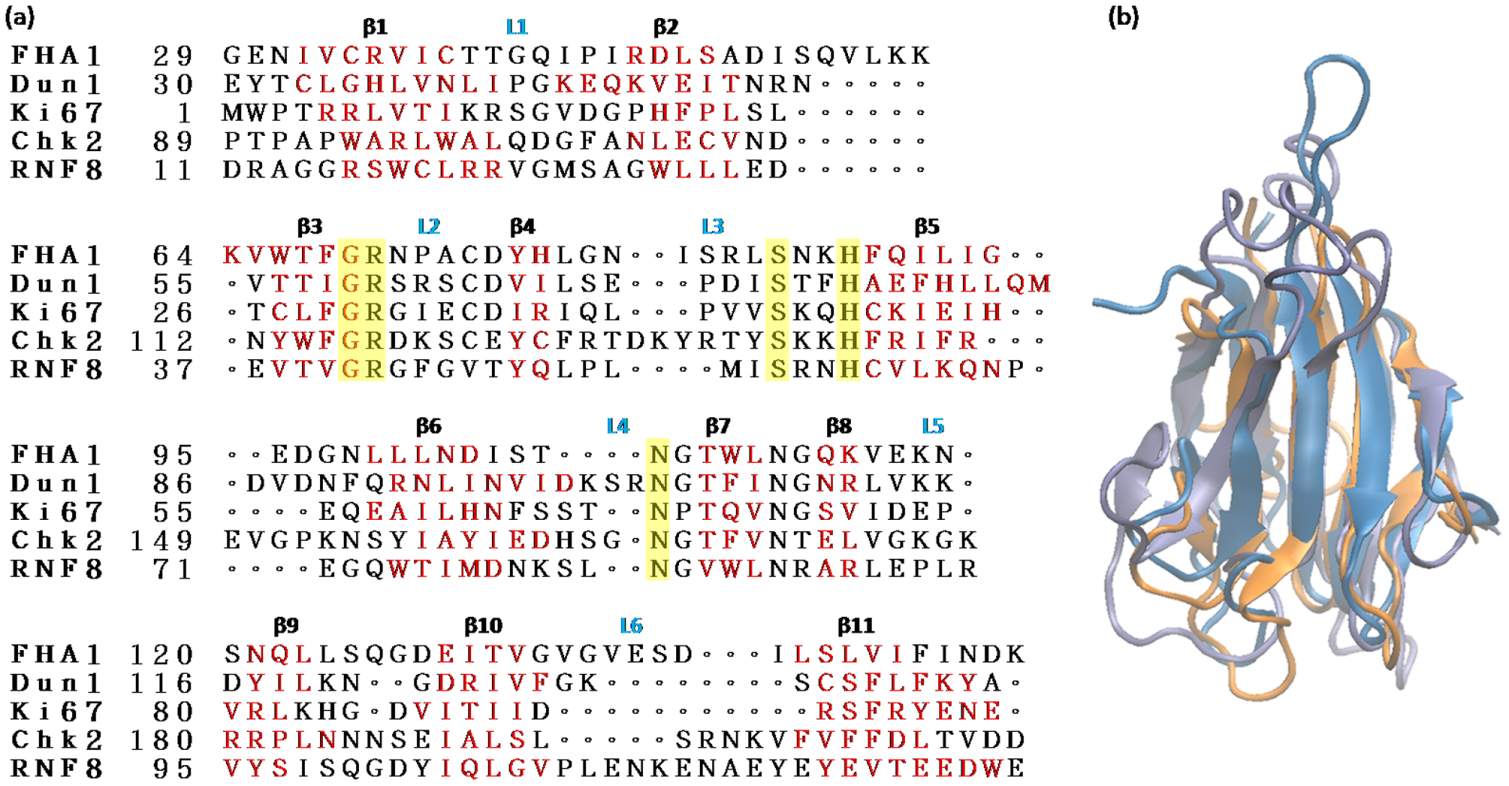
Sequence and structure alignment of the forkhead-associated (FHA) domain. (a) Sequence alignment of the FHA domain from different proteins. The β strands and loops are in red and black letters, respectively. The five conserved residues are highlighted in yellow. The detailed locations of the five conserved residues are in Figure 3. (b) Structure alignment of Rad53-FHA (gray), Dun1-FHA (blue) and Ki67-FHA (orange) domains.

**Figure 2:**
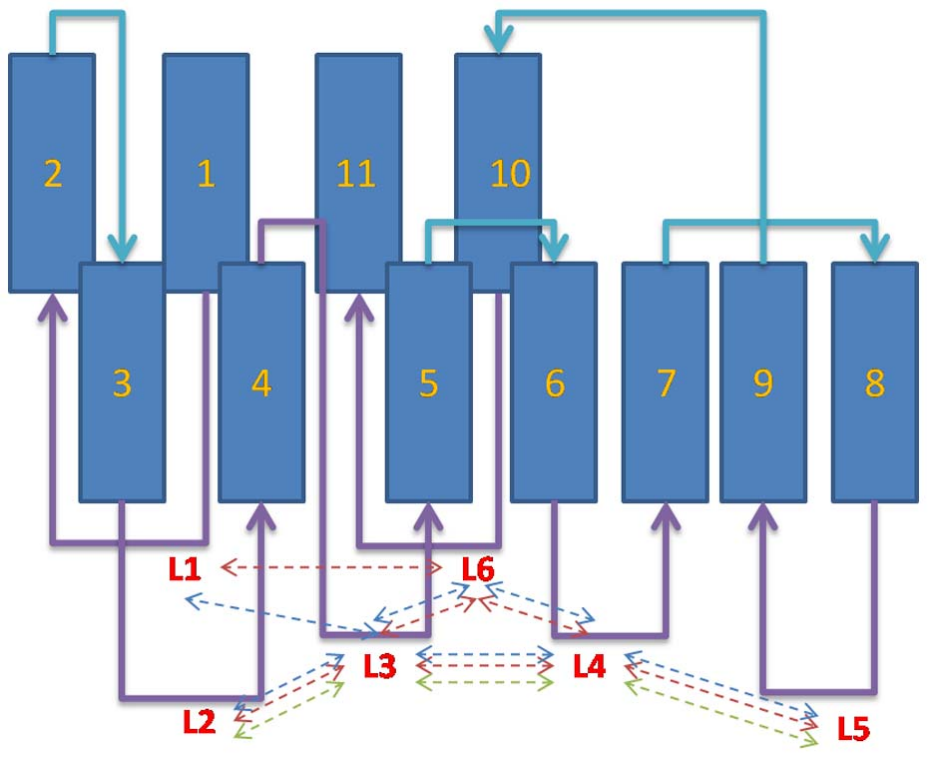
Topology of the FHA structure. The fold of the FHA domain includes 11 β sheets linked by loops. The loops that connect different β sheets are in purple and cyan lines. The sidechains of loops form a well-organized network by hydrogen bonds or van der Waal interactions to stabilize the domain. The red, blue and green dashed lines indicate loop interactions between labeled loops in Rad53-FHA1, Dun1-FHA and Ki67-FHA domains, respectively.

**Figure 3:**
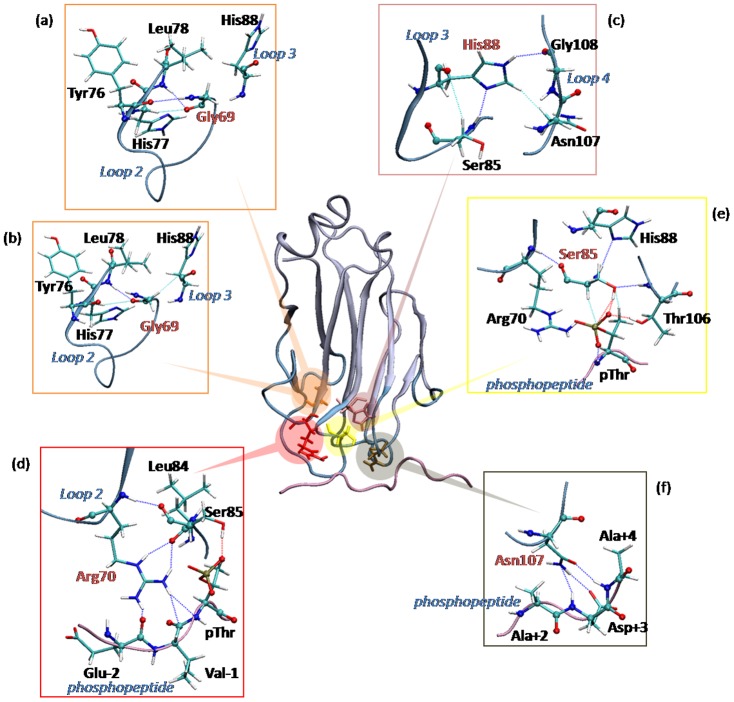
The detailed interactions of the five conserved residues. FHA domains contain five conserved residues, Gly (a) and (b), His (c), Arg (d), Ser (e) and Asn (f). Each conserved residue is shown in red letters. Blue and pink represent loops and peptides, respectively. The sidechain atoms are shown in bond representation. Red, blue and green dash lines indicate H-bond, salt-bridge and van der Waals interactions, respectively. This structure is taken from a molecular dynamics (MD) snapshot of the Rad53-FHA1 complex.

In a short time, the past 10 years, almost 100 FHA structures from different protein families have been deposited in the protein data bank (PDB) from both NMR and X-ray studies. All FHA domains share similar structural characteristics. The domain spans approximately 100 amino acid residues to fold into a twisted β sandwich of two large β sheets; each sheet contains five and six β strands (Figure 1). Some FHA domains have α helical insertions between the loops connected to the secondary β strands. Past experiments indicated that the six loops, opposite to the joining of the N and C terminus, directly involve peptide binding [23]–[25]. We numbered the 11 well-defined β strands and 6 loops from β1 to β11 and L1 to L6, respectively (Figure 2). The fluctuations in the loop region are the primary difference between different FHA domains. Although FHA domains adopt a similar topology fold, the spectrum of sequences is widespread. Sequence alignment revealed only five mostly conserved residues located in the binding loops or at the end of the β strand (Figure 1(a) and 3). These five conserved residues are typically considered a support for phosphopeptide recognition [21].

Despite sharing low sequence identity, FHA domains perform their function by grasping specific pThr substrates. Previous studies showed that the phosphate group of peptides interacts with the conserved and non-conserved residues around loops 2, 3 and 4 via hydrogen bonds, salt-bridges or both to form charge attractions [23], [26]. The binding discrimination between pThr and phosphoserine (pSer) can be attributed to the methyl group of pThr. This non-polar sidechain acts as a key that can nicely fit in the small cavity created by the conserved His, and the local contacts are stabilized by favorable van der Waals interactions between pThr and loops 3 and 4 [27]–[28]. Loops are typically flexible, but FHA domains show specificity for pThr via different loop orientations. Unlike other domains such as the BRCT and WW domains, whose binding regions usually involve a secondary structure such as α-helix or β-sheet, FHA domains are unique in that the domain–peptide interactions occur only around the loop surface [23], [28]–[29]. Although loops are typically considered a highly flexible region of a protein, work by Ding *et al.* indicated that the KI-FHA domain from kinase-associated protein phosphates (KAPPs) was natively rigid on the binding subsite. According to ^15^N NMR relaxation of nanosecond-scale motions, the fluctuations of the loop region were reduced, whereas the KI-FHA domain conferred peptide binding. The residues involving peptide recognition were also less movable in both free and bound states [24], [30]–[31].

To date, diverse FHA domains have been widely reported. The first structure of the Rad53-FHA1 domain in complex with the target peptide Pad9 was solved by Durocher *et al.* [21]. Both library screening and X-ray structure analysis revealed the Rad53-FHA1 domain with strong selectivity toward Asp in the +3 position (the third position after pThr residue) because of the critical role of Arg83 in the loop 3 [21], [23]–[24], [26], [32]. Later, the reported NMR structures suggested that in addition to Asp+3 binding mode, the Rad53-FHA1 domain binds to the pTXXI motif of the Mdt1 protein and pTXXT sequence of Rad53 protein, with X indicating any amino acid [33]–[34]. In contrast to the Rad53-FHA1 domain recognizing a single phosphorylated peptide, the Dun1-FHA domain interacts with Rad53-SCD1 peptide, which contains two pThr resides [34]. Moreover, the known structure of the Ki67-FHA domain complex with a 44-residue fragment of hNIFK is another example illustrating the promiscuity of FHA domains. The FHA domain of Ki67 antigen protein binds only to the extended sequence and fails to interact tightly with short phosphopeptides [35]. The above features suggest that FHA domains are highly plastic in respective ligands.

Although experimental studies have provided information about protein flexibility and rigidity, we still need to learn the correlation between dynamic movement and binding specificity or promiscuity. To address this question, computational works offer a powerful tool for deep insights [36]–[41]. For example, Huggins *et al*. studied phosphopeptide binding of the Polo-Box domain through molecular dynamics (MD) simulations, energy calculations and fluid solvation theory. The authors concluded that a phosphoresidue could generate major interactions with domain and water molecules to stabilize the charge of the phosphate group at the domain–peptide interface [39]. Basdevant *et al.* studied thermodynamic properties of protein–peptide interactions in the PDZ domain systems. The binding of the PDZ domain and peptide was mainly driven by favorable non-polar attractions. The entropic and dynamic aspects also play an important role in recognition [38].

Our goal in this study was to understand how signaling domains achieve peptide binding specificity and promiscuity via the structure of loops. We performed all-atom MD simulations with multiple FHA modules to capture domain motions and map the detailed conformational changes before and after peptide binding, including the system of Rad53-FHA1, Dun1-FHA and Ki67-FHA domains. To obtain the best knowledge of loop dynamics in the binding site, we carefully inspected the fundamental residues in loop–loop interaction networks. In addition, we examined the entropic contributions quantitatively to suggest the driving force of binding. Our work provides insights into how the intrinsic dynamic properties of a domain act as a transducer in response to promiscuous peptide recognition.

## Methods

### Molecular systems

We selected the free FHA domain from three different protein families, Rad53, Dun1 and Ki67, to study domain motions in a non-bound state. The initial coordinates were taken from the PDB codes 1G3G, 2JQJ and 1R21 [34], [42]–[43]. The Rad53-FHA1 domain binds to diverse peptide sequences. The protein–ligand complexes with the substrate binding peptide of Rad9 protein were obtained from the PDB codes 1G6G and 1K3Q, solved by X-ray and NMR, respectively [21], [26]. Another two bound Rad53-FHA1 structures, obtained from the PDB codes 2A0T and 2JQI, were in complex with the peptide Mdt1 and Rad53 protein, respectively [33]–[34]. The structure of the Dun1-FHA domain complex was acquired from the PDB code 2JQL, which corresponded with the di-phosphorylated peptide from the Rad53-SCD1 domain to activate Dun1 [34]. We also studied the Ki67-FHA domain complex. The initial structure with the optimal 44-residue fragment of phosphorylated hNIFK was explored by the coordinates from the PDB code 2AFF [43]. The details of the substrate peptide sequence from different proteins are in Table 1.

**Table 1:** Phosphopeptide sequences of FHA domain–peptide complexes.

Domain	Protein	PDB ID	Method	Phosphopeptide	Kd(µM)	ref.
**FHA1**	**Rad53**	1G6G	X-ray	LEV(**pT**)EADATFAK^†^	0.53	21
**FHA1**	**Rad53**	1K3Q	NMR	SLEV(**pT**)EADATFVQ^†^	0.3	26
**FHA1**	**Rad53**	2A0T	NMR	NDPD(**pT**)LEIYS*	15	33
**FHA1**	**Rad53**	2JQI	NMR	NI(**pT**)QPTQQST*	10	34
**FHA**	**Dun1**	2JQL	NMR	NI(pT)QP(**pT**)QQST*	0.3–1.2	34
**FHA**	**Ki67**	2AFF	NMR	KTVD(pS)QGP(**pT**)PVC(pT)***PTFLERRKSQVAE***LNDDDKDD*EIVFK*QPISC*	0.077	42

Sequences forming a secondary structure, α helix and β sheet, are in bold italic and italic format, respectively. Bold highlights the primary phosphothreonine binding residue. ^†^and * represent peptides from library screening and biological study, respectively.

### Molecular dynamics simulations

To study the protein dynamics in free and bound states, we performed MD simulations for non-bound FHA domains and FHA–phosphopeptide complexes using the Amber10 and NAMD2.6 simulation package [44]–[45]. The standard simulation procedures with Amber force field 99sb (ff99sb) were used for all processes [46]–[47]. Because phosphorylated amino acids were not included in the original ff99sb parameters, the pThr and pSer force field reported by Homeyer *et al.* were applied [48]. We performed 50-ns MD simulations for FHA systems including three free FHA domains and six domain–phosphopeptide complexes. We assigned the protonation states of the FHA domain by using the MCCE program [49]–[50]. All structures were solvated in a rectangular box of 12 Å TIP3P water by the tleap program in the Amber10 package; each system had about 40000 atoms [51]. Counter-ions of Na^+^ were placed on the basis of the Columbic potential to keep the whole system neutral. We also used Particle Mesh Ewald (PME) to consider the long-range electrostatic interactions [52]. After preparing 10,000 and 20,000 steps for water and system energy minimization, respectively, we gradually heated all systems from 250K for 20 ps, 275K for 20 ps and 300K for 200 ps. The resulting trajectories were collected every 1 ps with the time step 2 fs in an NPT ensemble. We applied the Langevin thermostat with a damping constant 2 ps^−1^ to maintain the temperature of 300K, and the hybrid Nose-Hoover Langevin piston method was used to control the pressure at 1 atm. The SHAKE procedure was used to constrain hydrogen atoms during MD simulations [53]. For post-MD analysis, we considered only 2- to 50-ns MD trajectories to ensure that all structures were in full equilibrium. The VMD program was used for visualization and graphical notation [54]. The MutInf script was used to capture correlated motions [55]. We also computed configurational entropy S for each dihedral angle by use of the T-Analyst program, with the Gibbs entropy formula as follows:



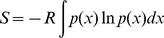
(1)where p(x) is the probability distribution of dihedral x, and R is the gas constant [56]. We considered only the internal dihedral degree of freedom of each dihedral, and the coupling between dihedrals was ignored. The change in configurational entropy between the free and bound state can be presented as follows:



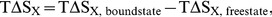
(2)where X denotes each dihedral angle, such as phi, psi, omega and sidechain.

## Results

We aimed to deeply understand how modular domains can exhibit various molecular recognitions with similar folding and how a slight change in loop structure leads to the binding of numerous partners. To study the domain motions of analogous architectures, we performed MD simulations on the FHA domain from different protein families, Rad53, Dun1 and Ki67. We chose these systems because both free protein and complex structures were available and they bound to novel motifs that had rarely been observed in other signaling domains. We used MD simulations with FHA complexes, free proteins, and proteins and peptides from complexes to investigate the dynamics of protein–peptide systems in the free and bound state.

### Investigation of force field and MD simulation results

Although MD simulation has been a common way to study protein dynamics, the choice of force field is always a key issue in initiating a correct simulation. The ff03 and ff99sb parameters are widely used in the Amber package [57]–[58]. However, as compared with the experimental structures, ff03 parameters cannot be used to create a correct model of the peptide C-terminus. The ensemble of the peptide backbone at the C-terminus tends to overstabilize the helical structure during simulations. We also observed that the important salt bridge between sidechain atoms of Arg83 and Asp+3 of the phosphopeptide was missing (Figure S1). These results disagree with crystal and NMR structures. In contrast, use of ff99sb allows for reproducing experimental structures and key interactions between the protein and phosphopeptide. Therefore, we used the ff99sb Amber parameters for all-atom MD simulations.

To confirm that simulations could reach a steady state, we checked the root mean square deviation (RMSD) shown in Figure S2 and considered the trajectories from only 2 to 50 ns for post-investigation. Moreover, we performed MD simulations on multiple initial structures from the same NMR ensemble. The domain motions remained consistent between different initial coordinates, and the important interactions between the peptide and domain held, although the peptide N- and C-termini were flexible. Hence, the MD simulations were independent of the initial structure selected from the NMR ensemble. In addition, in comparing two MD trajectories of the Rad53-FHA1 domain solved by X-ray and NMR, both showed similar motions during the simulation runs; therefore, we concluded that both structures obtained by X-ray and NMR study could offer a reasonable initial coordinate for MD simulation.

### Loop interaction networks of the FHA domain

Although not all loops of the FHA domain interact with the peptide directly, the interactions formed between the six FHA loops could be in charge of peptide binding. We observed that the residues around the loop region formed an extensive network through hydrogen bonds, salt bridges or van der Waals attractions. From MD trajectories, we could identify loop interaction networks from different FHA domain families (Figure 2). The key residues related to loop correlations around the binding area are in Table S1. The structural topology of different FHA domains is similar, but they show diverse loop interaction networks, which also affect the binding affinity of peptides.

In general, loops 3 and 4 are major pThr differentiated loops; loops 2, 5, and 6 are cooperative loops, stabilizing the whole system and balancing the remaining peptide sequence. For the Rad53-FHA1 domain, the binding loops 3 and 4 interact directly with pThr. These two loops also interact with loops 2, 5 and 6 to form a symmetric structure. Thus, the FHA domains feature a well-defined loop region relative to other signaling-related modules. Moreover, the loop interactions of the Dun1-FHA domain are similar to those of Rad53-FHA1; the only exception is loop 1. Like Rad53-FHA1, the Dun1-FHA domain uses two loop interactions (loops 3 and 4) via conserved residues Ser, Ser+1 and Asn-1 for pThr residue recognition.

Compared to Rad53-FHA1 and Dun1-FHA, the Ki67-FHA domain shows different interactions on the binding surface. We did not observe loop–loop interactions between loops 1 and 6, 3 and 6, and 4 and 6; instead, we found interactions between loops 2 and 3, 3 and 4, and 4 and 5 (Figure 2). Although the key residues in the primary pThr binding site are similar to that for Rad53-FHA1 and Dun1-FHA, the Ki67-FHA domain enlarges the distance of two large β sheets to assist the binding of the β strand from the long peptide. The largest difference between Ki67-FHA and the other FHA domains is that the Ki67-FHA domain has a short sequence in loop 6, which helps weaken the contacts between loop 6 and other binding loops. These alterations effectively take away the interactions of two large β groups and reveal the unique open-palm conformation. We successfully observed how FHA domains demonstrate alternative molecular recognitions by reassembling the loop relationship adjusted by sequence modifications.

### The role of conserved residues of the FHA domain

Although FHA domains have low sequence similarity (see Text S1 for details), they feature five highly conserved residues: Gly at the end of β3, Arg in the beginning of loop 2, Ser and His of loop 3, and Asn of loop 4 (Figure 1, highlighted in yellow). Arg, Ser and Asn bind directly to phosphopeptides; Gly and His interact with other residues of the FHA domain to stabilize the entire structure. Although the role of conserved residues has been discussed by observing direct interactions from crystal structures [21], here we studied the dynamics of conserved residues and attempted to discover how the conserved residues recruit peptides and display specificity in FHA recognition. The key interactions of each conserved residue are in Figure 3.

The conserved Gly plays an important role in loop 2 architecture. In aligning different FHA domains, the structure, shape and length of loop 2 is highly conserved as compared with other loops, such as loop 6. Moreover, Gly is located at the end of β3, which becomes a conjunction to aid communication between loops 2 and 3 (Figure 3(a) and (b)). The conserved His serves as a linkage between two pThr residue recognized loops, loops 3 and 4 (Figure 3(c)). The dynamic motions show that nitrogen atoms of His can form interactions with residues in both loops 3 and 4, which augments the loop communication. The conserved His is also directly related to pThr discrimination [28]. The conserved Arg and Asn include a charged sidechain. These sidechain atoms can form stable polar interactions with backbone atoms of a phosphopeptide (Figure 3(d) and (f)). Because Arg and Asn can directly interact with the peptide mainchain, fluctuating peptide sequences would not weaken binding affinity significantly. In addition, pThr is recognized by the conserved Ser (Figure 3(e)). As well as the FHA domain, other phospho binding domains such as the WW domain and BRCT repeats bind to phosphoresidues via one key Ser because the phosphate group can generate stable charge interactions with the hydroxide group of Ser [28], [59]–[60]. Therefore, the Ser sidechain can create proper attractions to connect phosphoresidues. Other details of the five conserved residues are in Text S2.

### Flexibility and rigidity of the FHA domain

**Figure 4:**
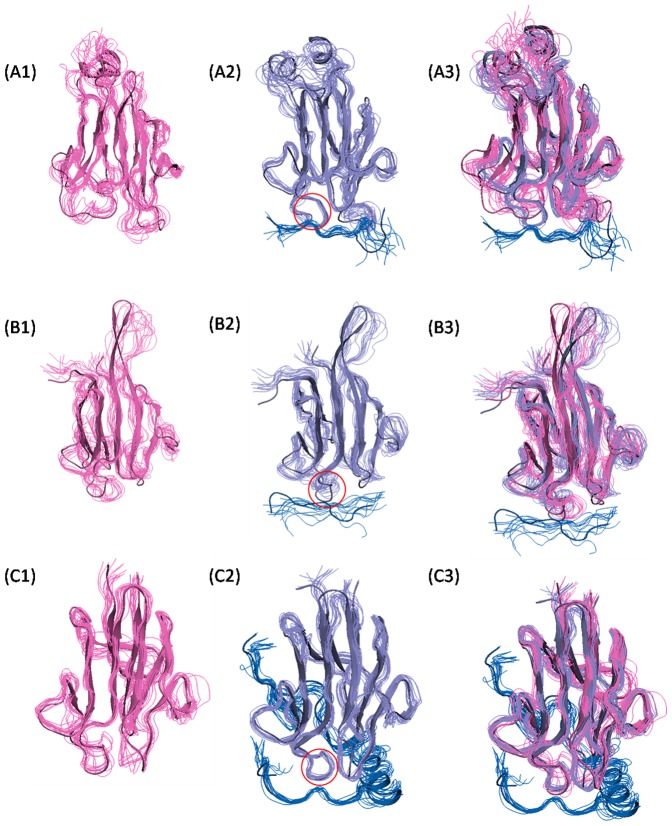
Superposition of MD snapshots. The trajectories from MD simulations show the flexibility of Rad53-FHA1 (A), Dun1-FHA (B) and Ki67-FHA (C) domains. We simulated the free domain (1) and complex (2) and superimposed the free and bound domain (3) to show the structural changes after the peptide bound. Pink, purple and blue represent free domain, bound domain and peptide, respectively. The pThr-binding loop 3 of the FHA domain is circled.

**Figure 5:**
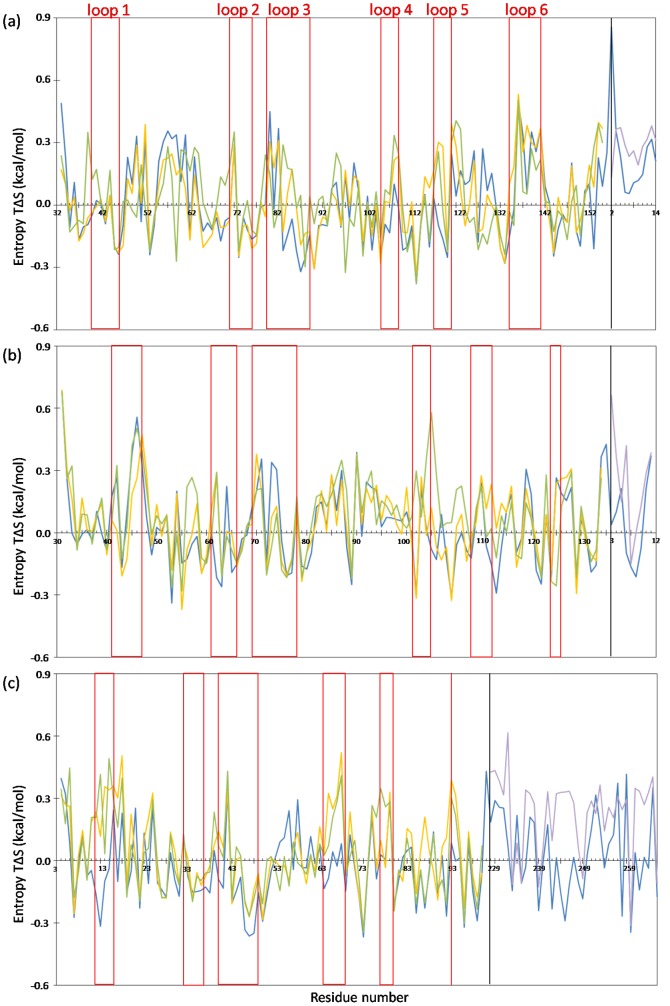
Entropy of the phi dihedral angle. The entropy calculations of the phi angle of the Ras53-FHA1, Dun1-FHA and Ki67-FHA domains are in (a), (b) and (c). Green, yellow, blue and purple represent free domain, domain from the complex, complex and free peptide, respectively. Red squares indicate six loops of each FHA domain. The region on the left and right of the black line indicate the domain and peptide region, respectively.

Figure 4 shows the dynamic ensemble of the FHA domain during 50-ns MD simulations. Although loops in most proteins are flexible, the FHA loops form a well-organized network and correlations via sidechain interactions to keep loops moderately rigid. This rigidity can help recruit peptide partners by reducing the entropy loss during the formation of a complex. To best understand FHA domain motions in the free state, we used T-Analyst to quantitatively calculate the changes in dihedral entropy of two free domains: the apo domain directly obtained from the PDB and the domain from the complex structure [56]. In general, the domain from the complex was slightly less flexible than the free domain, which suggests that the complex domain may not be fully relaxed (see green and yellow line in Figure 5). (The total entropy of the phi angle of the domain from the complex and free domain is 3.0 and 3.6 kcal/mol, respectively.) Then, we further studied domain motions before and after peptide binding. Figure 5 shows that the dynamics of the domain backbone do not change substantially after peptide binding. As well, the computed configurational entropy shows that the FHA domains are pre-organized in the free state (Table S2). The superposition of the free and bound domain is in Figure 4 (A3/B3/C3) and implies that the overall conformations of the FHA domain do not change substantially between the free and bound state. The entropy changes also confirm the results from correlation study (see next section), which indicates that the backbone hold structure and the sidechain play a role in loop correlations and peptide recognition.

On checking the entropy of the phi and psi torsion angle in the free FHA domain and complex, for Rad53-FHA1, the entropy did not change considerably in loops 1, 2 and 6 after peptide binding, but the pThr binding loops 3 and 4 showed significant entropy loss; especially, the pThr contact residues are more rigid in the bound than free state (see green and blue line in red boxes in Figure 5(a)). The conserved and key peptide-recognized residues of the Ki67-FHA domain are in general similar to those of Rad53-FHA1; however, loops 1 and 6 showed significant entropy loss because the peptide extensive surface bound to these two loops (Figure 5(c)). Although binding helped to keep the interaction surface rigid, loop 3 is more flexible in the bound Dun1-FHA domain (see the red circle in Figure 4(B2), compared to the red circles of Figure 4(A2) and 4(C2)); even the pThr contact residues, Ser74 and Thr75, do not show apparent entropy loss (Figure 5(b)). (The phi entropy loss of Ser74 and Thr75 is 0.14 and 0.10 kcal/mol, respectively.) Therefore, the flexible loop 3 destabilizes the recognition pocket of the primary pThr, and the Dun1-FHA domain needs the second phosphoresidue from the peptide to stabilize the entire complex.

### Correlated loop movements of the FHA domain in the free and bound state

**Figure 6:**
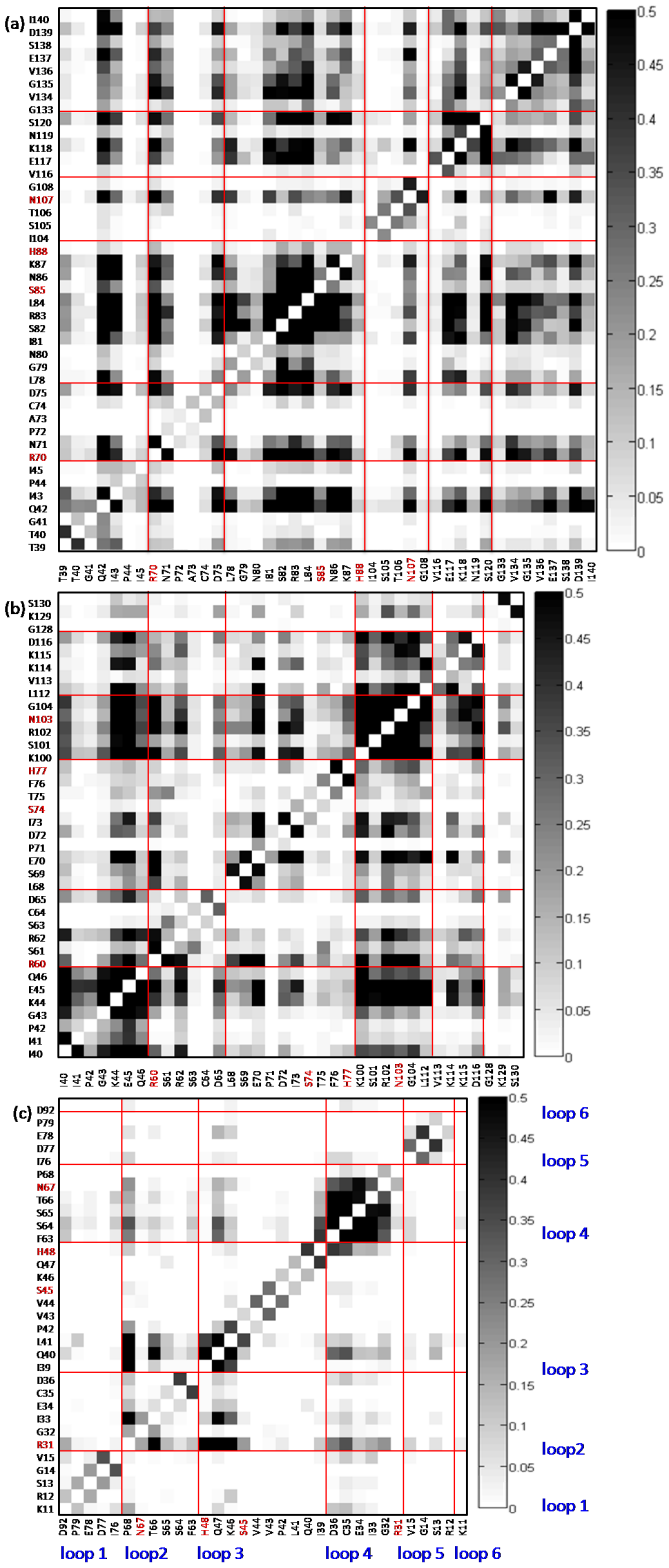
Correlation map of free FHA domain. The three FHA domains, Rad53-FHA1, Dun1-FHA and Ki67-FHA, are shown in (a), (b) and (c), respectively. We extracted the residues of 6 loops. The columns separated by red lines represent 6 loops of each FHA domain. For example, loops 1 to 6 is shown from the left to right column (see blue letters). Red letters are the conserved residues. The darker color means the two residues have stronger correlated movements (Black indicates strong correlations and white weak correlations.).

The correlated movements of subsites in a protein could show how the protein subunits relate to each other. Although MD simulations show that the binding loops can form an interaction network with links to each other, they do not show how the loops work together. For example, some residues do not form any chemical bonds, but they can move mutually. To identify the correlated movements of loops, we used the program MutInf to quantify the correlated movements between loops [55]. We can compare the correlation maps of Rad53-FHA1, Dun1-FHA and Ki67-FHA domains in both free and bound states (Figures 6 and S3) to understand the changes in correlated movements before and after peptide binding. Overall, the movements of the six loops in all FHA domains were correlated before and after peptide binding. Although the magnitude of correlated movement fluctuates, these cooperative loop movements help define the recognition site and maintain a particular structure for binding.

Unveiling the cooperative loop movements can help explain the diverse binding modes in different FHA systems. By checking correlation maps, we can understand whether one residue has more roles in the loop cooperation or peptide binding. The Rad53-FHA1 domain has well-correlated loop movements. Although loops 1, 5 and 6 do not directly contact the peptide, the co-movements of these three loops help stabilize the whole domain structure and create a potential surface for peptide access. The pThr binding residues Ser85 and Thr106 and conserved residue His88 show very weak correlations in movement (see the column of Ser85, Thr106 and His88 in Figure 6(a)). Therefore, these three residues play a role in pThr recognition instead of loop communication. The Ki67-FHA domain reveals similar correlated movements as Rad53-FHA1. The only difference is in loop 6. Movements of Asp92 of the loop 6 are not correlated with that of other loops (the column for loop 6 is white in Figure 6(c).), which is attributed to the β strand of the peptide in the Ki67 complex inserted between loops 1 and 6. However, the Dun1-FHA domain shows different correlations from those of Rad53-FHA1 and Ki67-FHA. The movements of the primary pThr recognition residues, Ser74, Thr75, His88 and Arg102, are correlated with that of other residues of the domain in the bound state (Figure S3(b)) instead of co-moving with pThr, which weakens the binding affinity of the primary phospho binding site in Dun1-FHA.

### Peptide recognition

**Figure 7:**
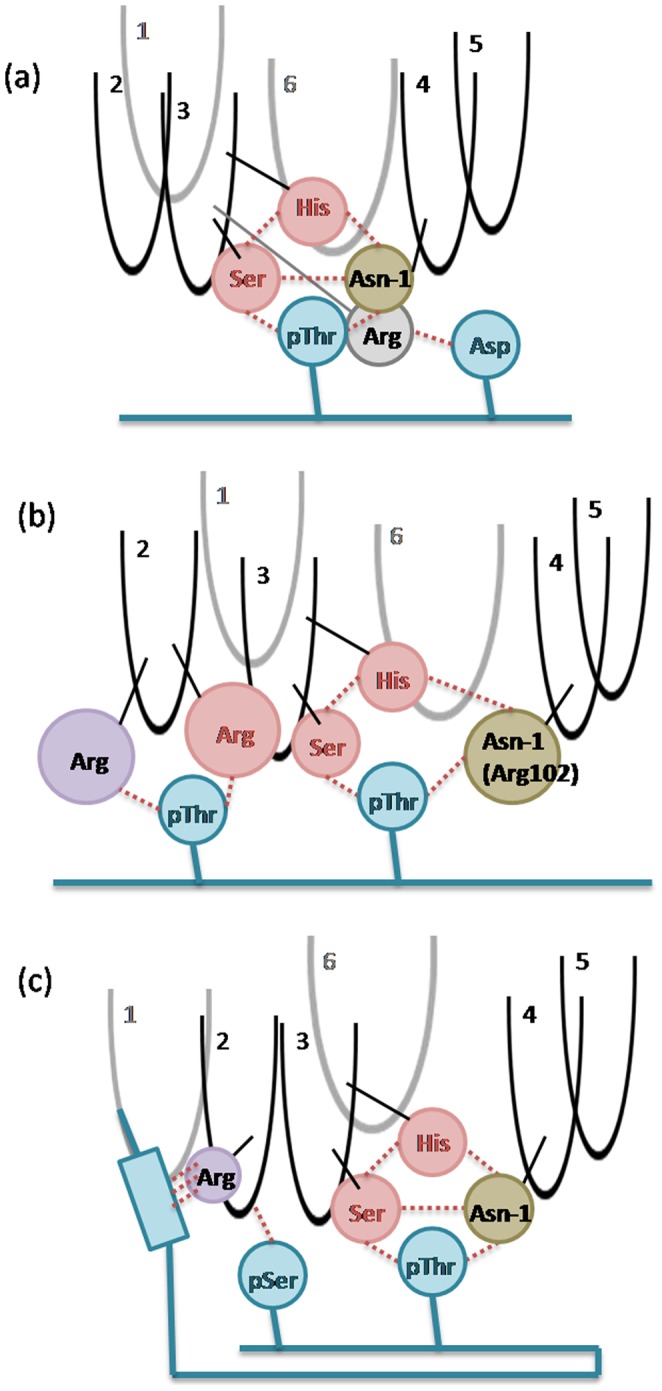
Cartoon of FHA–phosphopeptide binding mode. We summarize the key domain–peptide interactions of Rad53-FHA1 (a), Dun1-FHA (b) and Ki67-FHA (c) complexes. Black and gray shows loops in the front and at the back. Red and blue circle represents conserved residues and residues from the phosphopeptide, respectively. The brown circle shows the residue in front of the conserved residue Asn. The purple circle shows one Arg residue at loop 2. Red dash lines indicate possible interactions.

The FHA domain is a pThr binding module. The phosphate group of pThr can generate proper charge attractions and van der Waals interactions with the residues around the binding loops 2, 3 and 4. As compared with the primary pThr binding mode, the selectivity of pThr+3 is controversial, although the FHA pThr+3 rule of ligand recognition has been discussed [21], [32]. For the Rad53-FHA1 complex, the +3 pocket could access Asp, Ile or Thr through charge attractions, hydrophobic contact or both. The clear preference for Asp is due to the attractions between Asp+3 and Arg83 of loop 3 (Figure 7(a) and S4(A)). However, in the Dun1-FHA system, Ser at pThr+3 position failed to generate strong interactions with Asp; the Ki67-FHA domain does not show strong selectivity for pThr+3 either. In the RNF8-FHA system, non-polar residues Ile, Met and Leu located at Ser-1, Ser-2 and Ser-3 could form a hydrophobic pocket to include a non-polar substrate [61]. This observation explains why RNF8-FHA domains prefer a Tyr or Phe residue at pThr+3. Although the pThr+3 rule has been considered a useful way to search for potential biological partners, there is no apparent selectivity for this position because the binding is affected by an extensive recognition surface. Also, the implicit interactions between Ser-2 and pThr+3 are not enough to determine the binding mode.

Dun1-FHA domains possess di-pThr specificity, which raises the question of why Dun1-FHA is activated by two phosphoresidues. Our simulations show that the primary pThr binding site lacks stability to interact with the phosphate group perfectly. The dihedrals of the pThr sidechain rotate easily in the cavity of loops 3 and 4 because the huge sidechain of Arg102 locates between loops 3 and 4, which enlarges the distance between these two loops (Figure 4(B2), 7(b) and S4(B)). Thus, the binding pocket formed by loops 3 and 4 failed to provide a proper space for the methyl group of pThr. Another interaction between the second pThr and loop 2 helps to strengthen the binding. Two Args of loop 2 can form charge attractions to clip the phosphate group of the second pThr (Figure 7(b)). Accordingly, double phosphorylation is required for Dun1 activation. In addition, the dynamics of the Ki67-FHA domain from our simulations are in good agreement with experimental data. Other details of peptide recognition are in Text S3.

## Discussion

### Conserved pThr specificity in the FHA domain

Biological events typically require strict regulation via protein phosphorylation strategies. Phosphodomains can initiate signal transduction by forming multiprotein complexes. The common phosphoresidue binding domains include FHA, BRCT, WW, 14-3-3, and SH2 [29],[62]–[63]. The phosphate group is traditionally thought to generate robust charge attractions to anchor the specific binding surface effectively. In general, a few variant residues are near the phospho binding site in most signaling domains. For the BRCT domain, for example, residues in the pSer binding site include Ser/Thr-Gly in the β1/α1 loop and Thr-X-Lys motif at the N-terminus of α2, which are conserved in all pockets in different BRCT families, such as BRCA1, MDC1, PTIP and BARD1 [64]–[66]. This situation may erroneously suggest that the sequences in the phospho binding region diverge less. However, structural-based sequence alignment has revealed that FHA domains show only ∼25% conserved residues in the phospho recognition site as compared with other signaling domains (e.g., ∼100% and ∼60% conserved residues in the phospho binding area of the BRCT and WW domains, respectively [23], [64], [72]). Unlike most phospho binding modules, in addition to key electrostatic attractions of phosphoresidue recognition, FHA domains uniquely recruit the methyl group of pThr by van der Waals interactions, which provides a structural explanation for the pSer inactivation in the FHA domain.

### The power of loops controlling recognition

Binding by loop structure is a common method of modular domain recognition. As early publications illustrated, a phospho-related domain such as SH2 and a non-phospho binding module, PDZ, carry out specific protein–protein interactions mediated by forming deep loop binding pockets to harbor an exclusive motif [67]–[68]. Although the sequences vary around the loop region, the binding still can be engaged via charge and hydrophobic interactions. For the SH2 domain, loops control the accessibility of binding pockets; they can be plugged or opened for peptide recognition via a conformational switch by the EF and BG loop in various SH2 domains [69]. Moreover, the structures revealed that the PDZ domain specifically recognizes the peptide C-terminus. The terminal carboxylate group of a short segment binds to an extended groove between the second α helix (αB) and the second β strand (βB), containing the Gly-Leu-Gly-Phe sequence. This linkage loop defines a small cavity to confer a peptide sidechain [68]. In addition to the SH2 and PDZ domains, FHA domains show a unique loop working system. The peptide binding regions of FHA domains involve only loops, whereas both SH2 and PDZ domains bind to their partners through a single loop combined with at least one β strand or α helix. Our computational studies and post-analysis indicated that the molecular recognition of the FHA domain is principally through arrangements of six loops for grabbing a specific phosphate group and other coordinated residues around pThr. The different interactions and correlated movements of binding loops provide plasticity, which allows the region of a protein more flexible. This may result in promiscuous binding of the FHA domains. The finding also explains why FHA domains from different protein families can perform a selective and unique function by adjusting loop interactions.

In general, FHA domains bind to the protein sequences containing at least one pThr; however, each FHA member has a dissimilar preference for other residues surrounding the pThr. FHA domains can identify various sequences by using the analogous structural fold. This situation could be attributed to backbone atoms holding the overall homology, whereas sidechains define the binding surface toward a specific target. The steady interactions between 11 β strands are principally organized by a mainchain; thus, less mobility has been observed on the secondary β sheets. In contrast, the significant dynamics of the sidechain serves to accommodate the loop interface, so the optimal binding affinity can be maintained. As well, the sidechains at the FHA binding interface are more flexible than for other signaling domains such as the BRCT and WW domains, although the rigidity of the recognition site has been conventionally viewed as a common feature of the modular domain. Our data from entropy analysis provides additional insights into the comparison between the FHA and BRCT domain regarding the stability and flexibility of the binding site. Study of four BRCA1-BRCT complexes suggested small perturbations in both the conserved pSer-binding pocket and essential hydrophobic groove of the Phe clamp [40]. This entropic penalty constrains the motion of a flexible molecule, and the pre-aligned conformation might result in restricted peptide diversity, which explains why library screening could be easier for searching for potential binding targets of BRCT-containing protein. For other module domains such as SH2 and PDZ, as mentioned above, only one to three loops involve binding. Despite the plasticity of the binding loop, well-organized secondary structures, α helix and β strand, still limit possible recognition modes. Therefore, loop flexibility and networks increase the promiscuity of the FHA domain.

### Comparison of FHA peptide binding mode with other signaling domains

Protein recognition is a key element in regulating biological functions. Small domains of robust proteins are often responsible for these interactions. Typically, the signaling domain binds to its conjugated peptide in a two-pronged mode. For example, pTyr inserts into a positive charge pocket of the SH2 domain as the first binding subsite, and a hydrophobic residue at the C-terminus of peptide binds to a smaller groove as the second binding spot with a few additional interactions. Most SH2 domains exhibit specificity for hydrophobic affinity at pTyr+3, and some have residue preference for pTyr+2 and pTry+4 [14], [67]. The tandem BRCT repeats also adopt a similar binding mode; pSer is a charged anchor point, and one more hydrophobic residue at the +3 position is the other crucial plug [70]–[71]. Therefore, to fit two holes, the conformation of four amino acids from phosphoresidue to the +3 position becomes key for the substrate search. The domain scaffolds, which recognize a proline-rich peptide, are another common binding mode. WW domains can bind to a pThr-Pro– or pSer-Pro–containing motif [72]–[73]. The proline at C-terminal after the phosphoresidue could nicely fit a domain clamp and further restricts peptide conformations. SH3 domains use a similar structural strategy to interact with various proline-rich sequences, which can bend in a particular shape to dock into the binding groove [72], [74].

However, even if broad phosphopeptides have been tested by peptide screening, the binding mode of the FHA domain remains controversial. We lack knowledge of a clear relationship between structural features and the target molecule. Except for the conserved pThr specificity, no conserved second pronged mode exists equally in all FHA complexes because FHA domains include multiple loop subunits, and the assembly of loops increases recognition diversity. Therefore, FHA-domain–peptide interactions involve more residues and complicated peptide conformations. Although the strategy of the loop-adopting binding mode creates promiscuity, it does not lose the precision of the specific pThr recognition to regulate biochemical events in signaling networks.

## Conclusions

This work investigated how one domain can feature both binding specificity and promiscuity via loop structures. To address this question, we used the FHA domain as a model to study the loop interactions, correlated loop movements, conserved residues and flexibility/rigidity of domain binding loops. Our study suggests that the interactions and correlated movements between the six loops of the FHA domain play a pivotal role in defining the shape of the binding site. Despite variations in loop sequence and conformation of the FHA domain, a binding cavity could be open or closed for peptide recognition by switching the interactions and correlated movements of loop conformations. Although the various loop networks increase binding promiscuity, the specific recognition of the pThr is still held within different FHA families. The five conserved residues play key roles in domain structure or peptide recognition, The conserved Gly and His mediate the interactions of loops 2 and 3 and 3 and 4, respectively, whereas the conserved Ser, Arg and Asn interact with the phosphopeptide directly. Although loops in most protein systems are considered flexible, the FHA loops are moderately rigid. The rigidity of loops 3 and 4 help with the specific pThr recognition. The above features also explain the diverse binding modes of peptide recognition. For example, the Dun1-FHA domain requires a di-phosphopeptide for activation because of a flexible loop 3. The Ki67-FHA domain binds only to longer phosphopeptides because of correlated movement changes between loops 1 and 6. Our work provides insights into how molecular recognition can be achieved by loop arrangements to further help engineer potential peptide inhibitors.

## Supporting Information

Figure S1 **The comparison between Amber 03 and 99sb force field.** Purple and pink indicate the simulation of the FHA domain by applying ff99sb and ff03 force field; blue and orange indicate the simulation of the phosphopeptide from ff99sb and ff03 force field, respectively. (a) The snapshots are taken during 50 ns MD simulations. The thick blue and orange represents the peptide coordinate at 0 ns (after minimization and equilibrium). We observed that the peptide C-terminus tends to form a helical structure in ff03 simulation. (b) The snapshot at 0 ns (after minimization and equilibrium). The key residues, Arg83, Asp+3 and Thr+5 are shown in bond form. (c) and (d) show the conformation simulated by ff99sb and ff03, respectively. Salt-bridges and H-bonds are colored in blue and red, respectively.
(TIF)

Figure S2 **The root-mean-square-deviation (RMSD) plot.** Blue, red, green and orange represents Rad53-FHA1 (PDB: 1G6G), Rad53-FHA1 (PDB: 1K3Q), Dun1-FHA (PDB: 2JQL) and Ki67-FHA (PDB: 2AFF), respectively.
(TIF)

Figure S3 **Correlation map of Rad53-FHA1 (a), Dun1-FHA (b) and Ki67-FHA (c) bound domain.** We extracted only loop region here. The columns separated by the red lines represent six loops of each FHA domain. Red letters are the conserved residues.
(TIF)

Figure S4 **The key domain-phosphopeptide interactions of Rad53-FHA1 (A), Dun1-FHA (B) and ki67-FHA (C) complex.** We showed the overall structure in (1) and detail interactions in (2). Blue and pink indicates FHA domain and peptide, respectively. The dash lines show atom interactions.
(TIF)

Table S1 **List of significant residues related to loop-loop interactions from different FHA domain families.** Rad53-FHA1 and Dun1-FHA domain form well-organized loop interactions in both bound and free state; however, Ki67-FHA exhibit open-palm conformation due of lacking the interactions between two large β stands, therefore, the interactions between loop 3 and 6, 4 and 6, 1 and 6, and 1 and 3 disappear.
(DOCX)

Table S2 **The entropic changes before and after phosphopeptide binding.** TΔS_X_ = TΔS_X, bound state_ – TΔS_X, free state_.
(DOCX)

Text S1 Detail sequence alignments of signaling domains.
(DOCX)

Text S2 The five conserved residues of the FHA domain.
(DOCX)

Text S3 Details of peptide binding.
(DOCX)
