# Supplementary figures and images for: Achieving Peptide Binding Specificity and Promiscuity by Loops: Case of the Forkhead-Associated Domain
Source: PLoS One. 2014 May 28;9(5):e98291. doi: 10.1371/journal.pone.0098291 (PMC4037201; doi:10.1371/journal.pone.0098291)

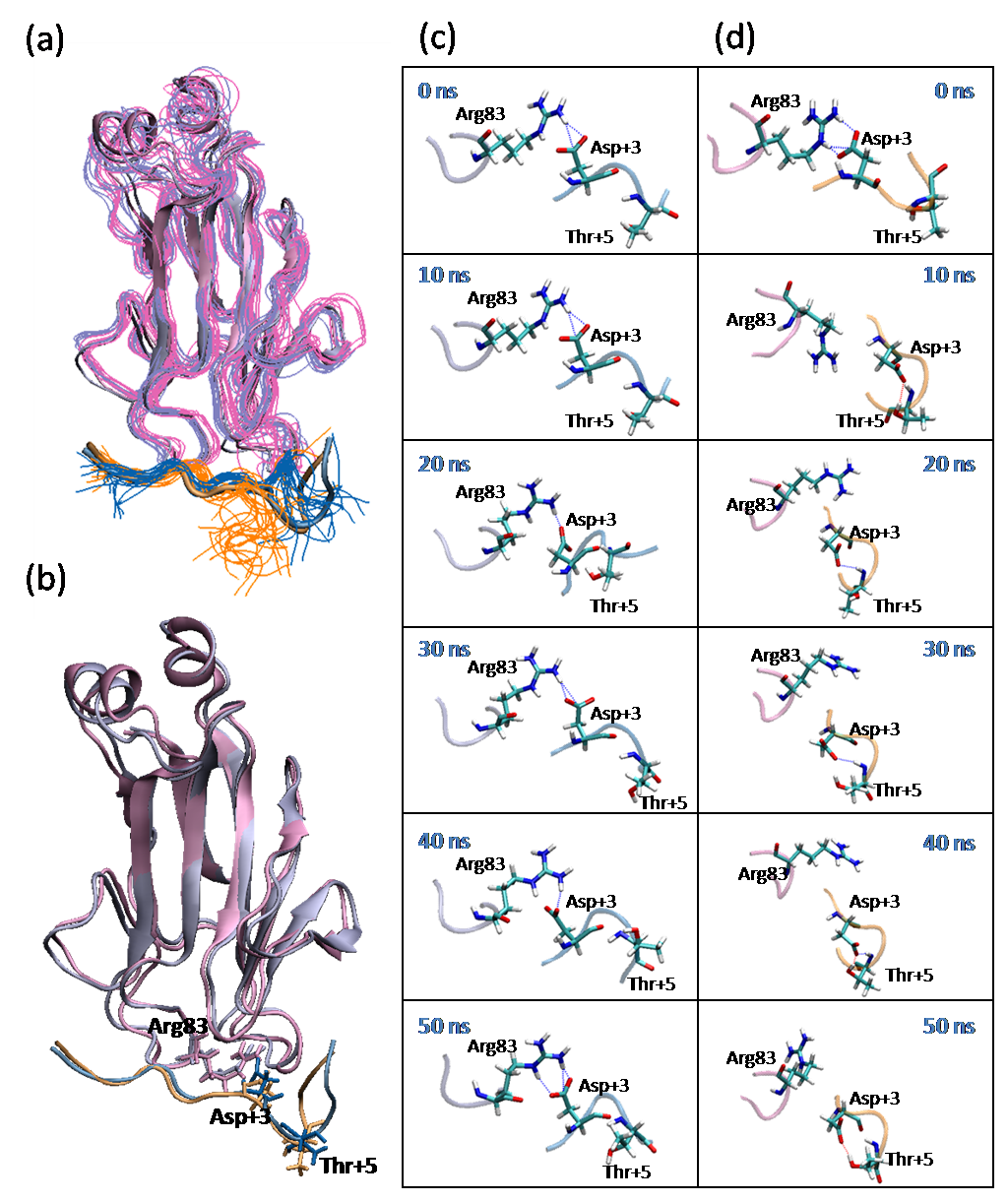

Supplement: Figure S1 — The comparison between Amber 03 and 99sb force field. Purple and pink indicate the simulation of the FHA domain by applying ff99sb and ff03 force field; blue and orange indicate the simulation of the phosphopeptide from ff99sb and ff03 force field, respectively. (a) The snapshots are taken during 50 ns MD simulations. The thick blue and orange represents the peptide coordinate at 0 ns (after minimization and equilibrium). We observed that the peptide C-terminus tends to form a helical structure in ff03 simulation. (b) The snapshot at 0 ns (after minimization and equilibrium). The key residues, Arg83, Asp+3 and Thr+5 are shown in bond form. (c) and (d) show the conformation simulated by ff99sb and ff03, respectively. Salt-bridges and H-bonds are colored in blue and red, respectively. (TIF) [file pone.0098291.s001.tif]

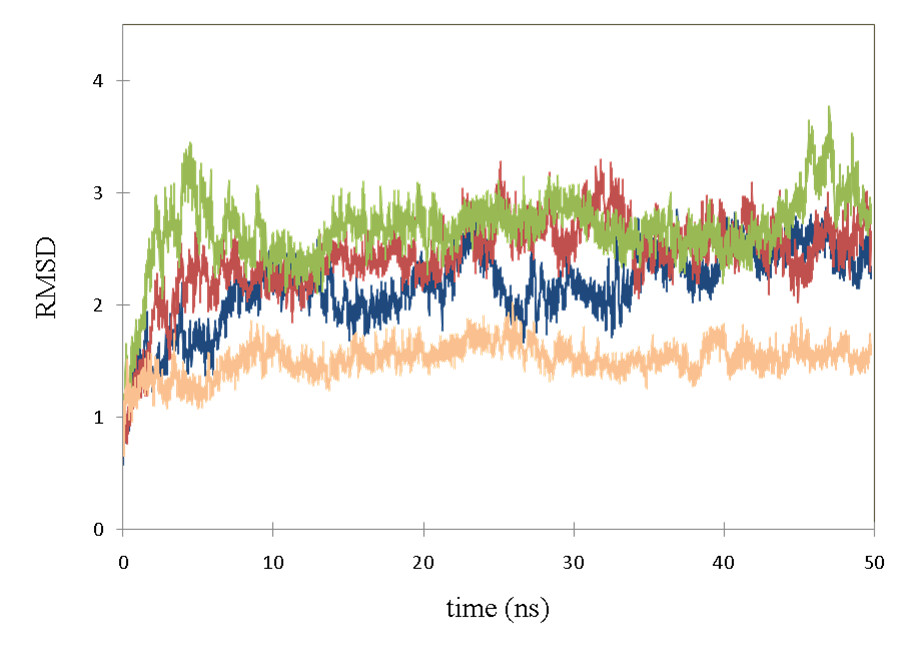

Supplement: Figure S2 — The root-mean-square-deviation (RMSD) plot. Blue, red, green and orange represents Rad53-FHA1 (PDB: 1G6G), Rad53-FHA1 (PDB: 1K3Q), Dun1-FHA (PDB: 2JQL) and Ki67-FHA (PDB: 2AFF), respectively. (TIF) [file pone.0098291.s002.tif]

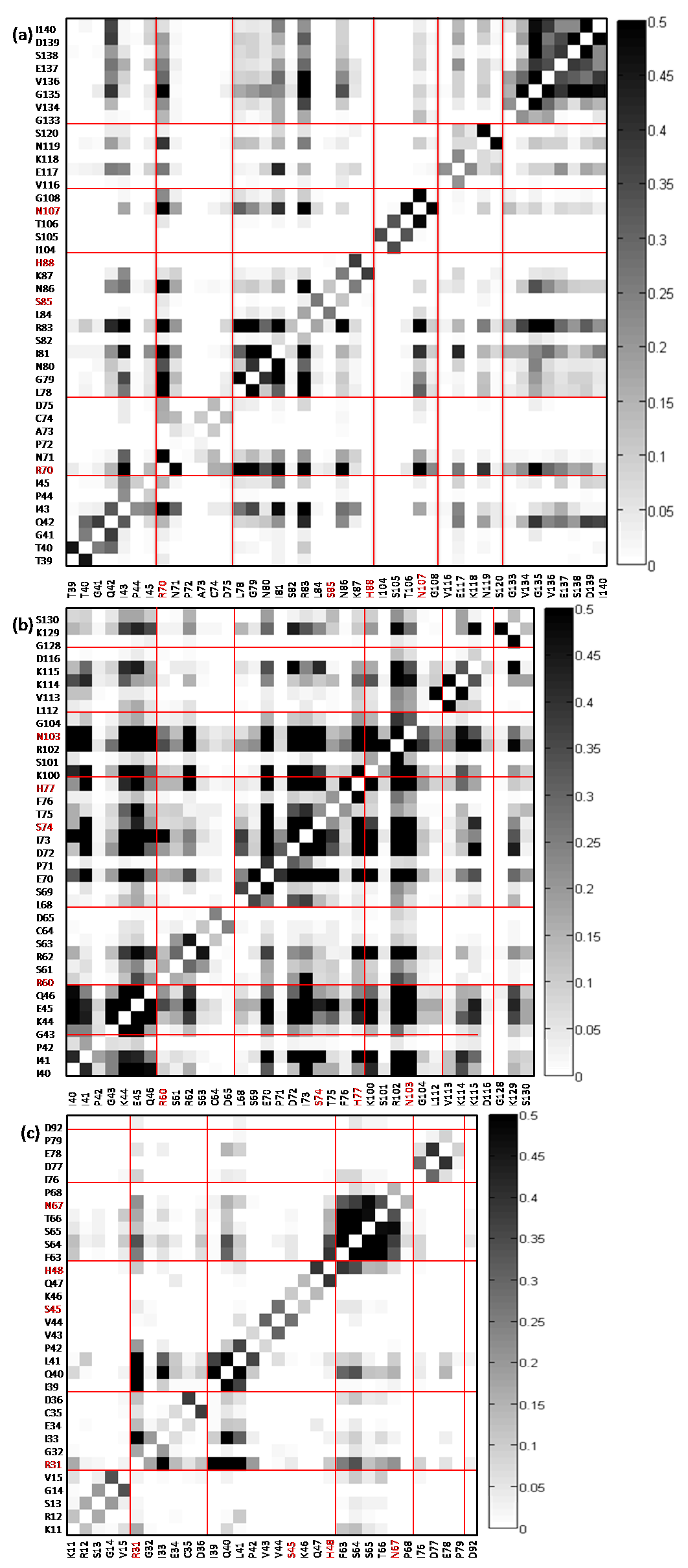

Supplement: Figure S3 — Correlation map of Rad53-FHA1 (a), Dun1-FHA (b) and Ki67-FHA (c) bound domain. We extracted only loop region here. The columns separated by the red lines represent six loops of each FHA domain. Red letters are the conserved residues. (TIF) [file pone.0098291.s003.tif]

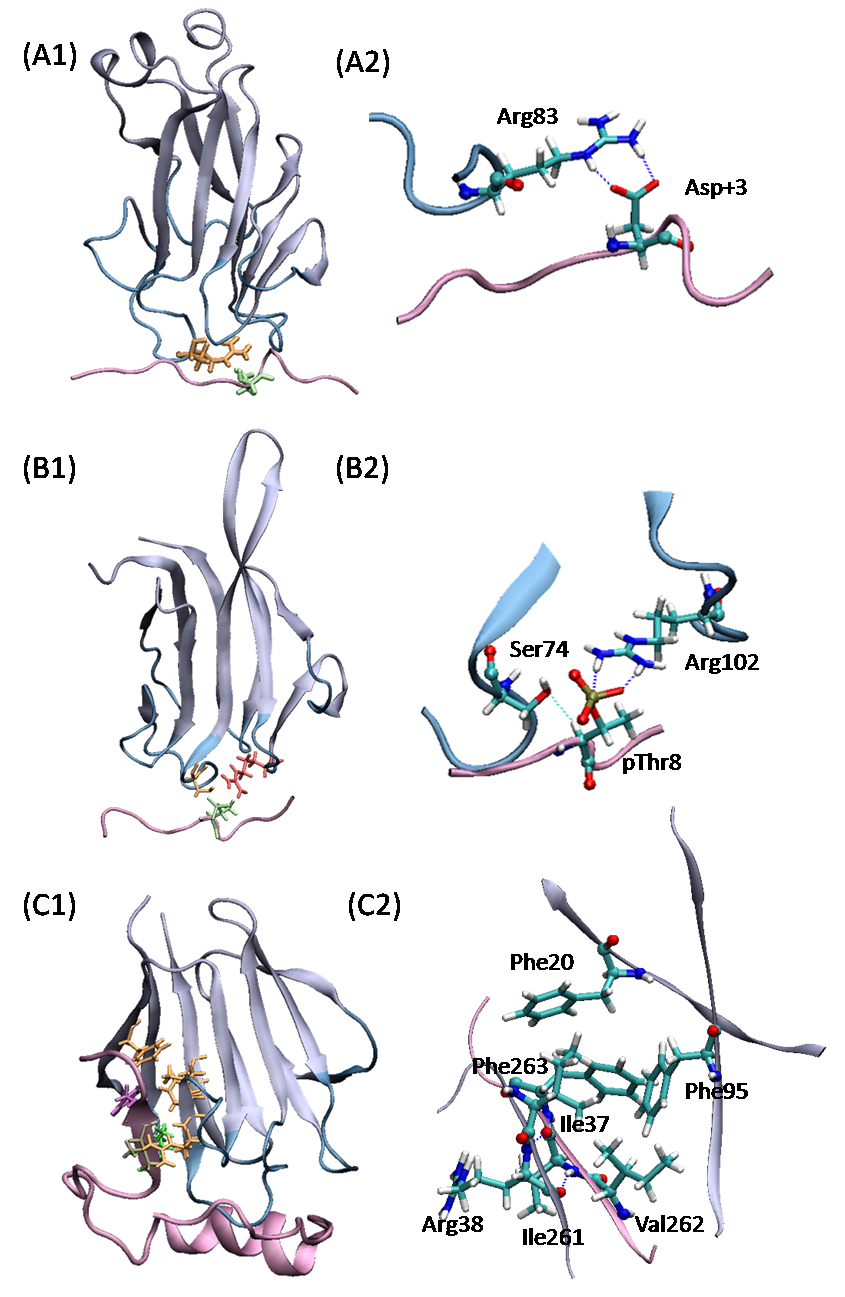

Supplement: Figure S4 — The key domain-phosphopeptide interactions of Rad53-FHA1 (A), Dun1-FHA (B) and ki67-FHA (C) complex. We showed the overall structure in (1) and detail interactions in (2). Blue and pink indicates FHA domain and peptide, respectively. The dash lines show atom interactions. (TIF) [file pone.0098291.s004.tif]
