# Supplementary material for: Achieving Peptide Binding Specificity and Promiscuity by Loops: Case of the Forkhead-Associated Domain
Source: PLoS One. 2014 May 28;9(5):e98291. doi: 10.1371/journal.pone.0098291 (PMC4037201; doi:10.1371/journal.pone.0098291)
Supplement: Table S1 — List of significant residues related to loop-loop interactions from different FHA domain families. Rad53-FHA1 and Dun1-FHA domain form well-organized loop interactions in both bound and free state; however, Ki67-FHA exhibit open-palm conformation due of lacking the interactions between two large β stands, therefore, the interactions between loop 3 and 6, 4 and 6, 1 and 6, and 1 and 3 disappear. (DOCX) [file pone.0098291.s005.docx]

|  | Rad53-FHA1 | Dun1-FHA | Ki67-FHA |
| --- | --- | --- | --- |
| loop 2 & loop 3 | R70, N71, I81, L84, S85, N86 | G59, R60, S69, D71, S74, T75 | R31, V44, S45 |
| loop 3 & loop 4 | R83, S85, T106, N107 | D72, I73, S74, R102, N103 | S45, H48, F63, S64, S65, P68 |
| loop 4 & loop 5 | N102, D103, S120, N129 | D99, S101, K115 | N62, D77 |
| loop 3 & loop 6 | S82, R83, G133, V134, D139 | E70, D72, K129, S130, C131 | none |
| loop 4 & loop 6 | N107, V134 | G104, G128 | none |
| loop 1 & loop 6 | T39, T40, Q42, I140, Q142 | none | none |
| loop 1 & loop 3 | none | K44, Q46, S69, E70 | none |
