# Supplementary material for: Achieving Peptide Binding Specificity and Promiscuity by Loops: Case of the Forkhead-Associated Domain
Source: PLoS One. 2014 May 28;9(5):e98291. doi: 10.1371/journal.pone.0098291 (PMC4037201; doi:10.1371/journal.pone.0098291)
Supplement: Table S2 — The entropic changes before and after phosphopeptide binding. TΔSX = TΔSX, bound state – TΔSX, free state. (DOCX) [file pone.0098291.s006.docx]

| domain | PDB ID | TΔS_phi_ | TΔS_psi_ | TΔS_omega_ | TΔS_total_backbone_ | TΔS_sidechain_ | TΔS_total_ |
| --- | --- | --- | --- | --- | --- | --- | --- |
| Rad53-FAH1 | 1G6G | -3.33 | -4.49 | -3.46 | -11.28 | -7.22 | -18.51 |
| Rad53-FAH1 | 1K3Q | -3.13 | -4.90 | -3.08 | -11.12 | -13.08 | -24.20 |
| Rad53-FAH1 | 2A0T | -3.62 | -3.65 | -1.85 | -9.13 | -6.17 | -15.90 |
| Rad53-FAH1 | 2JQI | -6.74 | -6.84 | -3.95 | -17.54 | -18.28 | -35.82 |
| Dun1-FHA | 2JQL | -5.13 | -9.23 | -1.68 | -16.05 | -10.89 | -26.94 |
| Ki67-FHA | 2AFF | -6.63 | -6.79 | -2.89 | -16.31 | -20.75 | -37.07 |
