# Supplementary material for: Achieving Peptide Binding Specificity and Promiscuity by Loops: Case of the Forkhead-Associated Domain
Source: PLoS One. 2014 May 28;9(5):e98291. doi: 10.1371/journal.pone.0098291 (PMC4037201; doi:10.1371/journal.pone.0098291)
Supplement: Text S1 — Detail sequence alignments of signaling domains. (DOCX) [file pone.0098291.s007.docx]

**Text S1**

In consideration of the entire domain sequence, the FHA domain has only ~4.7% conserved residues, however, the BRCT domain has ~30%, the WW domain has ~40%, and the PDZ domain has ~15% conserved residues. The details of sequence alignment of each domain are shown in the following reference.

FHA domain: Figure 4 of reference 23, DOI:10.1126/scisignal.151re12

BRCT domain: Figure 2 of reference 64, DOI: 10.1016/j.tibs.2004.09.010

WW domain: Figure 1 of reference 72, DOI: 10.1016/S0014-5793(01)03290-2

PDZ domain: Figure 2 of reference 67, DOI: 10.1016/S0092-8674(00)81307-0
