# Supplementary material for: Achieving Peptide Binding Specificity and Promiscuity by Loops: Case of the Forkhead-Associated Domain
Source: PLoS One. 2014 May 28;9(5):e98291. doi: 10.1371/journal.pone.0098291 (PMC4037201; doi:10.1371/journal.pone.0098291)
Supplement: Text S2 — The five conserved residues of the FHA domain. (DOCX) [file pone.0098291.s008.docx]

**Text S2**

*The conserved Gly*

Gly plays an important role for loop 2 architecture. Align different FHA domains, structure, shape and length of loop 2 are highly conserved compared to other loops such as loop 6. The nitrogen atom of Gly backbone forms salt-bridge with the oxygen atom of Gly+7 backbone. Also, the Gly backbone oxygen forms salt-bridge and van der Waals interactions with Gly+8 hydrogen and Gly+9 nitrogen atom. Because the interaction network is formed through atoms of backbone, the fluctuation of sidechain does not distort the loop 2 architecture. This structural motif also assists the stability of conserved Arg residue near Gly. Moreover, since the position of Gly is located at the end of β3, it becomes a conjunction to help the communication between loop 2 and 3. The weak van der Waals interactions between Gly and conserved His to hold the structure have also been observed. It is reasonable that Gly has smaller sidechain, otherwise, it may block the space between loop 2 and 3.

*The conserved His*

The conserved His is a linkage between two pThr recognized loops, loop 3 and 4. His is located at the beginning of β5, which helps to define the shape of binding site. Because of the shape and rich nitrogen atoms of His sidechain, the two stable salt-bridges are formed between His and conserved Ser or His and Asn+1 mainchain. The weak van der Waals attractions between His and conserved Asn have also been observed. Therefore, the importance of His sidechain is underlined through augmenting the communication of loop 3 and 4. Based on FHA sequence alignment, Asn+1 is usually the residue with smaller sidechain to reduce the block effect of communication between loop 3 and 4.

*The conserved Arg*

The conserved Arg residue has huge and charge sidechain, which forms stable salt-bridge with conserved Ser-1, pThr, pThr-1 and pThr-2 backbone atoms. Also, we observed the charge attractions between Arg sidechain and phosphate group of pThr. These contacts enlarge the interactions between domain and peptide and also explain why the mutagenesis of Arg to Ala reduces the binding affinity considerably. Moreover, the Arg residue forms solid interactions with conserved Ser to hold loop-loop correlations. The interaction networks between conserved residues, Gly, His, Arg and Ser are mainly through the contacts between backbone atoms, which define the loop shape around the peptide binding site. In Dun1-FHA complex, Arg has weaker interactions with standard pThr (pThr8); however, it forms stronger attractions with the other phosphoresidue, pThr5. Because Dun1-FHA domains need dual phosphate group to activate, the conserved Arg helps the second pThr recognition. In the case of Ki67-FHA complex, although Arg does not directly contact with the N-terminus phosphoresidue, pSer230, it forms good linkage with pSer-1 and pSer+1, which indicates that the conserved Arg shows a key role for peptide N-terminal recognition.

*The conserved Ser*

In addition to the linkage with conserved Arg and His, the conserved Ser forms salt-bridges with the conserved Asn, Asn-1 and Asn-2 through oxygen atom of Ser sidechain and loop 4 backbone. This connection is highlighted because the interactions between loop 3 and 4 define a small cavity to embrace the methyl group of pThr, which also responses to the selectivity of pThr from pSer. By far, it has been observed that the polar sidechain of Ser directly contacts with phosphate group. These stronger charge-charge attractions are the major interactions with phosphoresidue.

*The conserved Asn*

Unlike most conserved residues, which connect to each other and play a key role in structural issue, conserved Asn shows more feature in peptide recognition. The nitrogen and oxygen atom of Asn polar sidechain form stable interactions with peptide residue from pThr+1 to pThr+4. Because the conserved Asn directly interacts with peptide backbone, it allows FHA domains bind to peptide sequences with different sidechains. In other words, even the residues at peptide C-terminus fluctuate, it does not damage the binding affinity seriously. Taken together, the conserved Arg and Asn govern the recognition of peptide N- and C-terminus, respectively.
