# Supplementary material for: Achieving Peptide Binding Specificity and Promiscuity by Loops: Case of the Forkhead-Associated Domain
Source: PLoS One. 2014 May 28;9(5):e98291. doi: 10.1371/journal.pone.0098291 (PMC4037201; doi:10.1371/journal.pone.0098291)
Supplement: Text S3 — Details of peptide binding. (DOCX) [file pone.0098291.s009.docx]

**Text S3**

*Standard pThr recognition*

The standard pThr recognition is mainly contributed by conserved Ser, Ser+1 and Asn-1. The hydroxyl group of Ser sidechain could generate strong charge attractions with phosphate group of pThr. Then, Ser+1 residue forms interactions with pThr by salt-bridge of backbone NH or H-bonds of sidechain. Although the interactions between pThr and loop 4 are usually weaker than the attractions between pThr and loop 3, Asn-1 of loop 4 still makes contacts with pThr by charged interactions of sidechain (Figure 6(a)). Because the residue at Asn-1 position is not conserved among different FHA domains, the binding affinity between pThr and loop 4 relies on the sidechain of Asn-1. Therefore, the polar or charged residue at Ans-1 position may help pThr anchor.

*The pThr+3 specificity*

From library screening, Rad53-FHA1 domains display a preference toward Asp due to the attractions between Asp+3 and Arg83 of loop 3. Our simulations show that the pThr+3 residue can bind to conserved Asn and Ser-2, therefore, Ser-1 to Ser-3 of loop 3 play a key role on pThr+3 selectivity. In the complex of Rad53-FHA1 and phosphopeptide from Rad9, FHA domains prefer Ile residue to fit into +3 pocket through hydrophobic interactions with Gly135 and Val136 of loop 6 and Arg83 of loop 3. The non-polar residue, Leu+2 and Tyr+4, also make hydrophobic contacts with domain to strengthen binding. Moreover, Rad53-FHA1 also interacts with the peptide sequence from Rad53 itself. In this system, our results indicate that Thr+3 is surrounded by the residues of loop 3, 4 and 6. Hydroxyl group of Thr+3 sidechain directly contact with conserved Asn, Gly135 and Val136 backbone via charge attractions. The non-polar methyl group of Thr+3 play a role to fit into accessible space created by loop 3, 4 and 6 by van der Waals interactions, which is similar as Ile+3 of Rad9 peptide.

*The Ki67-FHA second phosphoresidue recognition*

Ki67-FHA domain does not show any preference to short peptide. The peptide extensive surface helps binding. Ile261, Val262 and Phe263 on the β-strand of hMIFK peptide interact with Thr9, Phe20, Ile33, Ile37, Arg38, Ile39 and Phe95 of FHA domain. These results are also in good agreement with experimental observation (Figure 6(c) and S5(C)). Except Val262 binds to Arg38 by forming salt-bridge, other sidechain atoms burry between β1 and β4 interact with each other through hydrophobic interactions. Because lacking direct interactions between loop 1 and 2 of free domain, β1, β2, β3, β4 and β11 form a hydrophobic pocket to recruit β-strand of extensive peptide in bound state. Although there is the second phosphoresidue, pSer230, near loop 2 of Ki67-FHA complex, the second phosphate group rotates away from the binding surface occasionally. This observation provides a reasonable explanation that the tight binding affinity is mostly contributed from extensive β-strand, not the pSer230.
